# The Use of Bruton Tyrosine Kinase Inhibitors in Waldenström’s Macroglobulinemia

**DOI:** 10.1007/s44228-022-00007-5

**Published:** 2022-05-23

**Authors:** Obada Ababneh, Hassan Abushukair, Aref Qarqash, Sebawe Syaj, Samer Al Hadidi

**Affiliations:** 1 Faculty of Medicine, Jordan University of Science and Technology, P.O. 22110, Irbid, Jordan; 2 Myeloma Center, Winthrop P. Rockefeller Cancer Institute, University of Arkansas for Medical Sciences, Little Rock, AR USA

**Keywords:** Waldenstrom’s Macroglobulinemia, Bruton tyrosine kinase inhibitors, Ibrutinib, Zanubrutinib

## Abstract

The use of Bruton Tyrosine Kinase (BTK) inhibitors in Waldenström’s Macroglobulinemia (WM) is evolving. Ibrutinib, a first-generation BTK inhibitor, is currently approved for use in frontline and relapsed/refractory disease. Second-generation BTK inhibitors are being used and studied to improve clinical outcomes and/or safety profile. Zanubrutinib, one such second-generation inhibitor, was recently approved in treatment-naive and refractory/relapsed patients. Here, we review the use of BTK inhibitors in WM in front-line and refractory or relapsed settings. We also highlight common adverse events, the emergence of BTK inhibitors resistance, and future directions of their use.

## Introduction

Waldenström’s Macroglobulinemia (WM) constitutes approximately 1–2% of all hematological malignancies. It is a rare type of non-Hodgkin lymphoma (NHL) that is more common in the elderly, with a median age of 70 years at diagnosis. It is characterized by infiltration of the bone marrow by monoclonal immunoglobin M (IgM) protein-producing lymphoplasmacytic cells [1, 2]. The incidence of WM is higher in males, older age, and non-Hispanic Caucasians [3]. One fourth of WM patients are asymptomatic at the time of the diagnosis and may remain symptom free without any treatment for several years [4, 5]. The median overall survival (OS) for symptomatic patients is approximately 9 years with the 10-year OS ranging from 8 to 84%, according to the revised IPSSWM classification [6].

Treatment of symptomatic WM depends on patients’ comorbidities and preferences, and availability of treatment options. Preferred primary treatment options, according to the National Comprehensive Cancer Network (NCCN) guidelines, include anti-CD20 monoclonal antibodies (rituximab) based regimens, either in combination with chemotherapy (chemoimmunotherapy, CIT) or proteasome inhibitors [7]. Chemoimmunotherapy can be associated with some toxicities and many patients will inevitably develop resistance and will require further lines of therapy [8].

Bruton Tyrosine Kinase inhibitors (BTKi) are used frequently in the treatment of WM. Ibrutinib was approved by the Food and Drug Administration (FDA) and is currently included as part of the preferred regimens in the NCCN guidelines for both treatment-naïve (TN) and relapsed/refractory (R/R) disease, either as a single agent or in combination with rituximab [7]. Zanubrutinib, a second generation BTKi, was recently approved by the FDA [9]. In this article, we review the pathogenesis of WM and the role of BTKi in its management both in front-line and R/R disease settings. We further discuss BTKi related adverse events (AE), treatment resistance and future directions.

## Pathogenesis of WM and Role of BTKi

The understanding of the role of specific cytogenetic alterations in WM has helped in identifying its pathogenesis [10]. The myeloid differentiation primary response 88 (MYD88) is an adaptor molecule partially activated by direct interaction with BTK, which results in a cascade of events that eventually lead to the release of nuclear factor Kβ p65 (NF-Kβ p65), which drives its prosurvival signaling [11–14]. Inhibition of MYD88 in vitro decreases the release of NF-Kβ and causes cytotoxicity and inhibition of cell growth [14, 15]. The presence of MYD88 somatic mutations is a characteristic feature in WM and may help in the diagnostic process. MYD88L265P is the most common mutation which exists in almost 90% of WM patients [16–18]. Mutated MYD88 has the ability to constitutively homodimerize and allow downstream signaling without receptor activation, which triggers prosurvival signaling via BTK, PI3K/AKT, and MAPK/ERK1/2 [19]. MYD88 can also drive prosurvival NF-Kβ and mTOR signaling by acting as part of a multiprotein supercomplex (My-T-BCR) formed by MYD88, Toll-like receptor 9 (TLR9), and B-cell receptor (BCR) [20].

BTK inhibition reduces NF-Kβ signaling and promotes apoptosis, making BTK a viable therapeutic target [19, 21, 22]. In addition to BTK, hematopoietic cell kinase (HCK) was found to be more activated and expressed in primary WM cells, which is triggered by IL-6 after over-expression of mutated MYD88. Knockdown of HCK is associated with reduced cell survival and attenuation of BTK, PI3K/AKT, and MAPK pathways. Inhibition of HCK by A419259 blocks the activation of HCK by IL-6 and induces apoptosis in WM cells and in activated B-cell diffuse large B-cell lymphoma (ABC DLBCL) cells. This makes HCK a potential therapeutic target to consider in the management of WM, either by itself, in combination with BTK inhibitors, or by a BTK/HCK dual inhibitor such as KIN-8194 [23, 24]. Ibrutinib has an off-target effect against HCK, while other BTKi have a reduced off-target effect [23, 25]. However, the efficacy of these agents was not inferior to ibrutinib in treating WM, as will be demonstrated in the next sections.

C-X-C chemokine receptor type 4 (CXCR4) is a G protein-coupled receptor (GPCR) that acts as a chemokine receptor when it binds to its ligand CXCL12, leading to chemotaxis, lymphocyte trafficking, cell cycle proliferation, migration, and stemness. It is the second most commonly mutated gene in WM, which can occur in approximately 50% of patients [26–29]. The CXCR4 somatic mutations in WM are identical to the germline variants found in a rare disease called WHIM syndrome (warts, hypogammaglobulinemia, infection, and myelokathexis), thus a mutated CXCR4 is denoted CXCR4WHIM [30, 31]. Patients with CXCR4WHIM mutation have reduced sensitivity to ibrutinib, more aggressive disease, higher degree of bone marrow involvement, higher IgM levels, hyperviscosity, all of which may be associated with shorter treatment-free survival [19, 32].

## Use in Frontline Settings

Most WM clinical trials using BTKi included patients with R/R disease. Characteristics and outcomes of clinical trials using BTKi in front-line settings in WM patients are summarized in Table 1. The use of BTKi in TN patients was assessed in 30 patients who received ibrutinib until progression or intolerable toxicity. After a median follow up of 4 years, a 100% objective response rate (ORR) with 87% major response rate (MRR), and 30% very good partial response (VGPR) or a complete response (CR) were reported [33]. Ibrutinib was studied in combination with rituximab in TN patients with an ORR of 91% [34, 35]. A study that compared ibrutinib, a first generation BTKi, with zanubrutinib, a second generation BTKi, showed a higher percentage of 18-month event free survival rate among TN patients treated with ibrutinib when compared to zanubrutinib, though with wide confidence interval (94% versus 78%; 95% CI 63–99 and 52–91, respectively) [34, 35].

**Table 1:** Characteristics and outcomes of clinical trials using BTKi in front line settings in WM patients

Study ID	Study Design	Intervention	TN sample (%)	Mutational status	Outcomes
Dimopoulos (2020) [36]	Phase 3	Zanubrutinib	5 (17.9)	MYD88WT/ CXCR4WT: 5	ORR: 80% MRR: 40% VGPR/CR: 20% 18-mo PFS: 60% 18-mo OS: 80%
Tam (2020) [35]	Phase 3	Ibrutinib	18 (18.2)	MYD88L265P/CXCR4WT: 17 MYD88L265P/CXCR4UNK: 1	ORR: 89% MRR: 67% VGPR/CR: 17% 18-mo PFS: 94%
		Zanubrutinib	19 (18.6)	MYD88L265P/CXCR4WT: 18 MYD88L265P/CXCR4WHIM: 1	ORR: 95% MRR: 74% VGPR/CR: 26% 18-mo PFS: 78%
Buske (2021) [34]	Phase 3	Ibrutinib + Rituximab	34 (45.3)	^a^MYD88L265P/CXCR4WT: 32 MYD88L265P/CXCR4WHIM: 26 MYD88WT/CXCR4WT: 11	ORR: 91% MRR: 76% VGPR/CR: 27%
Castillo (2021) [33]	Phase 2	Ibrutinib	30 (100)	MYD88L265P/CXCR4WT: 16 MYD88L265P/CXCR4WHIM: 14	ORR: 100% MRR: 87% VGPR/CR: 30% 48-mo PFS: 76% 48-mo OS: 100%
Owen (2019) [38]	Phase 2	Acalabrutinib	14 (13.2)	^a^MYD88L265P: 50	ORR: 93% MRR: 79% VGPR/CR: 0%
Sekiguchi (2020) [39]	Phase 2	Tirabrutinib	18 (66.7)	MYD88WT/CXCR4WHIM: 1 MYD88L265P/CXCR4WT: 13 MYD88L265P/CXCR4WHIM: 3	ORR: 94.4% MRR: 88.9% VGPR/CR: 16.7%
Trotman (2020) [37]	Phase 1/2	Zanubrutinib	24 (31.2)	MYD88L265P/CXCR4WT: 14 MYD88L265P/CXCR4WT: 4 MYD88L265P/CXCR4UNK: 2 MYD88WT/CXCR4WT: 3	ORR: 100% MRR: 87.5% VGPR/CR: 33.3% 24-mo PFS: 91.5% 24-mo OS: 100%

*TN* treatment naïve, *ORR* objective response rate, *MRR* major response rate, *VGPR* very good partial response, *CR* complete response, *PFS* progression-free survival, *OS* overall response, *WT* wildtype, mo months, UNK unknown

^a^Genotype for this study was for the entire cohort (TN + R/R)

A phase II trial of zanubrutinib showed a MRR of 87.5% and a 24-month OS of 100% in TN patients, with lower responses in patients with no MYD88 mutation (MRR: 40%, 18-month OS: 80%) [36, 37].

Acalabrutinib, a second-generation BTKi, showed a similar ORR, and MRR were observed, though no TN patients achieved VGPR/CR [38]. Tirabrutinib was studied on 18 TN patients with 94% ORR and 89% MRR [39].

## Use in R/R Patients

### First-Generation BTKi

In the pivotal phase II trial that led to the approval of the first BTKi in WM patients, ibrutinib was given until disease progression or intolerance in 63 R/R patients [40]. With a median follow-up of 59 months, an ORR of 91% with a VGPR/CR rate of 30% were observed, whereas the 60-month progression-free survival (PFS) was 54%. Previous treatment with 3 or more versus 1–2 lines of therapy was associated with a lower PFS (60-month PFS: 38% versus 68%, respectively, *P* = 0.01); yet, no significant association was found when comparing MRR or VGPR/CR rates [40]. In the iNNOVATE trial subgroup analysis, an ORR and MRR of 87% and 77%, respectively, were reported on 31 R/R WM patients on ibrutinib with a 60-month OS of 73% [41]. In that study, MYD88 mutated/CXCR4 wildtype patients had an 88% MRR compared to 71% in MYD88 mutated/CXCR4 mutated patients. When combined with rituximab, ibrutinib was found to have a slightly higher VGPR/CR of 34% in 41 R/R patients compared to other ibrutinib trials [34]. In ASPEN’s head-to-head comparison between ibrutinib and zanubrutinib the response and survival rates were similar (ORR: 94% versus 94%, MRR: 80% versus 78%, 18-mo PFS: 82% versus 86%, respectively) [35].

### Second-Generation BTKi

Zanubrutinib demonstrated a relatively high rate of VGPR/CR (51%) in 53 R/R patients in a phase I/II clinical trial in which 38 patients had the MYD88 mutation [37]. In contrast, in the sub-study cohort of ASPEN, zanubrutinib was only administered to MYD88 wild-type patients and resulted in relatively lower efficacy, with an ORR of 81% and a VGPR/CR rate of 29% [36]. Another phase-2 trial of zanubrutinib in R/R patients showed ORR of 77%, VGPR/CR rate of 33% and a 24-month PFS and OS of 60.5% and 87.8%, respectively [42].

Acalabrutinib was studied in 92 patients with R/R disease and produced an ORR of 93% with 9% VGPR/CR [38]. Tirabrutinib showed ORR of 100% and a MRR of 89% in R/R patients with the MYD88 mutation and wildtype CXCR4 [39]. Characteristics and outcomes of clinical trials using BTKi in R/R WM patients are included in Table 2.

**Table 2:** Characteristics and outcomes of clinical trials using BTKi in relapsed/refractory settings in WM patients

Study ID	Study Design	Intervention	R/R sample (%)	Mutational status	Outcomes
Dimopoulos (2020) [36]	Phase 3	Zanubrutinib	23 (82.1)	MYD88WT/CXCR4WT: 18 MYD88WT/CXCR4WHIM: 1 MYD88WT/CXCR4UNK: 2 MYD88UNK/CXCR4UNK: 2	ORR: 81% MRR: 52% VGPR/CR: 29% 18-mo PFS: 71% 18-mo OS: 90%
Tam (2020) [35]	Phase 3	Ibrutinib	81 (82.8)	MYD88L265P/CXCR4WT: 73 MYD88L265P/CXCR4WHIM: 8	ORR: 94% MRR: 80% VGPR/CR: 20% 18-mo PFS: 82%
		Zanubrutinib	83 (81.4)	MYD88L265P/CXCR4WT: 73 MYD88L265P/CXCR4WHIM: 10	ORR: 94% MRR: 78% VGPR/CR: 29% 18-mo PFS: 86%
Trotman (2021) [41]	Phase 3	Ibrutinib	31 (100)	MYD88L265P/CXCR4WT: 17 MYD88L265P/CXCR4WHIM: 7 MYD88WT/CXCR4WT: 1 Unavailable: 6	ORR: 87% MRR: 77% VGPR/CR: 29% 60-mo OS: 73% Median OS: NR
Buske (2021) [34]	Phase 3	Ibrutinib + Rituximab	41 (54.7)	^a^MYD88L265P/CXCR4WT: 32 MYD88L265P/CXCR4WHIM: 26 MYD88WT/CXCR4WT: 11	ORR: 93% MRR: 76% VGPR/CR: 34%
Treon (2020) [40]	Phase 2	Ibrutinib	63 (100)	MYD88L265P/CXCR4WT: 36 MYD88L265P/CXCR4WHIM: 22 MYD88WT/CXCR4WT: 4 Unavailable: 1	ORR: 90.5% MRR: 79.4% VGPR/CR: 30.2% 60-mo PFS: 54% 60-mo OS: 87%
Owen (2019) [38]	Phase 2	Acalabrutinib	92 (86.8)	^a^MYD88L265P: 50	ORR: 93% MRR: 80% VGPR/CR: 9%
Sekiguchi (2020) [39]	Phase 2	Tirabrutinib	9 (33.3)	MYD88L265P/CXCR4WT: 9	ORR: 100% MRR: 88.9% VGPR/CR: 0%
An (2021) [42]	Phase 2	Zanubrutinib	44 (100)	MYD88L265P/CXCR4WT: 32 MYD88L265P/CXCR4WHIM: 5 MYD88WT/CXCR4WHIM: 1 MYD88WT/CXCR4WT: 6	ORR: 76.7% MRR: 69.8% VGPR/CR: 32.6% 24-mo PFS: 60.5% 24-mo OS: 87.8%
Trotman (2020) [37]	Phase 1 /2	Zanubrutinib	53 (68.8)	MYD88L265P/CXCR4WT: 26 MYD88L265P/CXCR4WT: 7 MYD88L265P/CXCR4UNK: 5 MYD88WT/CXCR4WT: 8	ORR: 93.9% MRR: 79.6% VGPR/CR: 51% 24-mo PFS: 76.2% 24-mo OS: 91.5%

*R/R* relapsed/refractory, *ORR* objective response rate, *MRR* major response rate, *VGPR* very good partial response, *CR* complete response, *PFS* progression-free survival, *OS* overall response, *WT* wildtype, *mo* months, *UNK* unknown, *NR* not reached

^a^Genotype for this study was for the entire cohort (TN + R/R)

## BTKi Safety

The use of ibrutinib is associated with some AEs, which might be explained by its multiple inhibitory effects on different proteins such as EGFR, Src, ITK, TEC, and HCK [43]. The most common AEs are rash, fatigue, diarrhea, cytopenias, respiratory tract infections, bleeding, atrial fibrillation (AF), and hypertension (HTN). In the iNNOVATE trial, 19% of patients experienced any grade AF, and 16% of all patients suffered from grade 3/4 AF from a combination of ibrutinib and rituximab after a median follow-up of 60 months. However, the longer use of ibrutinib-rituximab did not increase the prevalence of grade 3/4 AF after the first 2 years of therapy (8% at 0–1 years, 6% at 1–2 years, and 9% at 3–5 years). A similar trend was also observed with grade 3/4 HTN after the first 3 years (27% at 0–3 years and 9% at 3–5 years) [34, 44]. History of AF was associated with earlier development of AF with a median of 4 months, whereas patients without such a history developed AF within a median time of 33 months [45, 46]. Previous systematic reviews confirmed the increased risk of AF, HTN, and bleeding events on ibrutinib [47–49].

Second-generation BTKi are more selective and produce lower off-target effect compared than ibrutinib [50, 51]. In the ASPEN trial, diarrhea, muscle spasms, peripheral edema, AF, and pneumonia were higher in the ibrutinib arm compared with the zanubrutinib [35]. Although grade 3/4 neutropenia were common in the zanubrutinib arm, the infection rate was similar between the two groups. Dose reduction was needed in 14% of patients under zanubrutinib and in 23% in the ibrutinib arm.

Acalabrutinib can be associated with headache (39%), diarrhea (33%), contusion (29%), dizziness (25%), fatigue (23%), nausea (23%), upper respiratory tract infections (22%), constipation (21%), and arthralgia (20%). The most common grade 3/4 AEs were neutropenia (16%), pneumonia (7%), anemia (5%), and lower respiratory tract infections [38]. Similar to zanubrutinib, only 5% of the patients developed AF.

Tirabrutinib’s most common AEs were rash (44%), neutropenia (26%), leukopenia (22%), stomatitis (15%) and thrombocytopenia (11%). Three patients required dose reduction due to bleeding events and one patient discontinued treatment due to atypical mycobacterial infection [39].

BTKi should be administered continuously until disease progression or severe toxicity, as patients who discontinued their treatment had a poor prognosis [52, 53]. In case of severe AEs, dose reduction and/or the use of other treatment without dropping the BTKi might be preferred. It should be noted that dose reduction resulted in improved or resolved AEs with no effect on treatment efficacy [54]. Table 3 summarizes some of the most important AEs across different BTKi.

**Table 3:** Summary of common adverse events in clinical trials using BTKi

Drug	Previous Therapy Status	Atrial Fibrillation	Infection^b^	Hypertension	Neutropenia	Anemia	References
Ibrutinib	R/R: 100%	All grade: 12.7% Grade 3–4: 1.6%	All grade: 27% Grade 3–4: 6.3%	All grade: 6.3% Grade 3–4: 0%	All grade^a^ (2–4): 23.8% Grade 3–4: 15.9%	All grade^a^ (2–4): 4.8% Grade 3–4: 1.6%	[40]
Ibrutinib + Rituximab	TN: 45% R/R: 55%	All grade: 19% Grade 3–4: 16%	All grade: NR Grade 3–4: 29%	All grade: 25% Grade 3–4: 15%	All grade: NR Grade 3–4: 9%	All grade: 19% Grade 3–4: 11%	[34]
Zanubrutinib	TN: 19% R/R: 81%	All grade: 2% Grade 3–4: 0%	All grade: 24% Grade 3–4: 10%	All grade: 11% Grade 3–4: 6%	All grade: 29% Grade 3–4: 20%	All grade: 12% Grade 3–4: 5%	[35]
Acalabrutinb	TN: 13.2% R/R: 86.8%	All grade: 5% Grade 3–4: 1%	All grade: 84% Grade 3–4: 23%	All grade: 5% Grade 3–4: 2%	All grade: 17% Grade 3–4: 16%	All grade: 10% Grade 3–4: 5%	[38]

*NR* not reported, *R/R* refractory/relapsed, *TN* treatment-naïve

^a^Only grade 2–4 adverse events were available

^b^Studies report detailed infectious events and as a result a patient could experience two or more infections (eg: pneumonia and urinary tract infection)

## Special Considerations

Currently, BTKi have been used in WM indefinitely with discontinuation only upon disease progression or intolerable toxicity. This approach could potentially increase the risk of acquired treatment resistance, as well as the occurrence of AEs. Alternative treatment schedules that include fixed duration of BTKi treatment, which is currently being investigated in chronic lymphocytic leukemia, can be further studied in the future [55, 56].

The use of BTKi can be associated with an IgM rebound phenomenon, which can manifest as symptomatic hyperviscosity, cold agglutinin disease, cryoglobulinemia, or peripheral neuropathy. The IgM rebound phenomenon is defined as a rise in IgM by 25% after treatment discontinuation, with an absolute increase of at least 5 g/L within three months after discontinuation of treatment in the absence of disease progression [52, 53]. BTK constitutively activates STAT5A and STAT5B, which increase IgM secretion in WM cells, which might explain the IgM rebound following ibrutinib discontinuation [57, 58]. In a retrospective study, 73% of the patients who discontinued ibrutinib had an IgM rebound [52]. In addition, 16% developed symptomatic hyperviscosity and required plasmapheresis. In another study, 60% of patients had an IgM rebound after ibrutinib discontinuation with 34% developing symptomatic hyperviscosity [59]. One study found that the median IgM level at the time of symptomatic hyperviscosity was 61.8 g/L (range 31–124 g/L) [60]. Thus, the abrupt discontinuation of ibrutinib even for disease progression or AEs should be avoided. Close monitoring of IgM levels after ibrutinib discontinuation is warranted. According to the consensus treatment recommendations from the 10th International Workshop for Waldenström’s Macroglobulinaemia, bridging therapy with ibrutinib in combination with the next-line of treatment should be considered before completely stopping ibrutinib [61]. Due to the increased risk of bleeding, it is recommended to suspend ibrutinib 3–7 days before surgery and resume treatment 1–3 days after the procedure [62].

Bing–Neel syndrome (BNS) is a rare condition with central nervous system involvement of WM cells [63]. There is currently no standard treatment of BNS, and agents with good central nervous system penetration can be considered [64]. A multicenter cohort study of 28 BNS patients treated with ibrutinib demonstrated a 5-year OS rate of 86% [65]. Other BTKi are used less frequently, with case reports showing some efficacy of zanubrutinib and tirabrutinib [66, 67].

The use of CIT strategies such as rituximab-dexamethasone-cyclophosphamide and bendamustine-rituximab have previously demonstrated substantial efficacy in WM [68, 69]. However, experience with CIT over BTKi or vice versa as primary therapy to treat WM is still lacking. There are no published head-to-head trials comparing CIT with BTKi in WM. Currently, the RAINBOW trial (NCT04061512) is comparing Rituximab-Ibrutinib with Dexamethasone-Rituximab-Cyclophosphamide in TN WM. Altered TP53 has been associated with chemoresistance in CLL [70, 71]. TP53 aberrations were associated with poor prognosis in one cohort where 78% were treated with a chemo-containing regimen in TN WM [28]. It has been suggested that ibrutinib can bypass TP53 mutation in WM cells [72]. Real-world data powered by next-generation sequencing revealed ibrutinib as optimal therapy in TN patients [73]. Taken together, patients with the MYD88 mutation regardless of CXCR4 and TP53 status are likely to benefit from BTKi-containing regimens. Clinical trials comparing BTKi with CIT should incorporate TP53 status to inspect this claim.

## Future Directions

Future studies should aim to address treatment options post BTKi therapy. Acquired resistance to BTKi is a challenge [74]. The use of daratumumab and veneteclax is currently under investigation (NCT02677324) [75]. Minimizing AEs related to BTKi is important, and this can be done by preferential use of either first or second generation BTKi according to patients’ comorbidities, and with more focus on quality of life [76, 77]. Ongoing studies are exploring the role of combination therapies that include ulocuplumab, a monoclonal antibody that inhibits the binding of CXCR4 to CXCL12 [78]. A recent phase I trial evaluated the combination of ulocuplumab-ibrutinib in WM patients with CXCR4 mutation, with preliminary results showing a good safety profile and an estimated 2-year PFS of 90% [79]. Mavorixafor is another highly selective anti-CXCR4, with a recent study in combination of ibrutinib that showed rapid and clinically meaningful reduction in IgM levels in WM patients with CXCR4 mutation [80].

The optimum salvage therapy after BTKi resistance is not established. The use of CIT in alkylator-based regimens, such as bendamustine-rituximab and dexamethasone-rituximab-cyclophosphamide can be considered as salvage therapy following ibrutinib [59]. A resent phase II trial showed promising efficacy of orelabrutinib in R/R WM [81]. Ongoing trials are testing next-generation, non-covalent reversible BTKi, such as pirtobrutinib (NCT03740529) and nemtabrutinib (NCT03162536) that bind to non-BTKC481. The development of effective agents for patients who progress on BTKi is undergoing.
